# The student resilience survey: psychometric validation and associations with mental health

**DOI:** 10.1186/s13034-016-0132-5

**Published:** 2016-11-03

**Authors:** Suzet Tanya Lereya, Neil Humphrey, Praveetha Patalay, Miranda Wolpert, Jan R. Böhnke, Amy Macdougall, Jessica Deighton

**Affiliations:** 1 Evidence Based Practice Unit (EBPU), UCL and Anna Freud National Centre for Children and Families, London, N1 9JH UK; 2 Manchester Institute of Education, School of Environment, Education and Development, University of Manchester, Manchester, UK; 3 Institute of Psychology, Health and Society, University of Liverpool, Liverpool, UK; 4 Mental Health and Addiction Research Group (MHARG), Hull York Medical School and Department of Health Sciences, University of York, York, UK; 5 Respiratory Epidemiology and Public Health Group, National Heart and Lung Institute, Imperial College, London, UK

**Keywords:** Resilience, School surveys, Mental health, Quality of life, Psychometrics

## Abstract

**Background:**

Policies, designed to promote resilience, and research, to understand the determinants and correlates of resilience, require reliable and valid measures to ensure data quality. The student resilience survey (SRS) covers a range of external supports and internal characteristics which can potentially be viewed as protective factors and can be crucial in exploring the mechanisms between protective factors and risk factors, and to design intervention and prevention strategies. This study examines the validity of the SRS.

**Methods:**

7663 children (aged 11–15 years) from 12 local areas across England completed the SRS, and questionnaires regarding mental and physical health. Psychometric properties of 10 subscales of the SRS (family connection, school connection, community connection, participation in home and school life, participation in community life, peer support, self-esteem, empathy, problem solving, and goals and aspirations) were investigated by confirmatory factor analysis (CFA), differential item functioning (DIF), differential test functioning (DTF), Cronbach’s *α* and McDonald’s *ω*. The associations between the SRS scales, mental and physical health outcomes were examined.

**Results:**

The results supported the construct validity of the 10 factors of the scale and provided evidence for acceptable reliability of all the subscales. Our DIF analysis indicated differences between boys and girls, between primary and secondary school children, between children with or without special educational needs (SEN) and between children with or without English as an additional language (EAL) in terms of how they answered the peer support subscale of the SRS. Analyses did not indicate any DIF based on free school meals (FSM) eligibility. All subscales, except the peer support subscale, showed small DTF whereas the peer support subscale showed moderate DTF. Correlations showed that all the student resilience subscales were negatively associated with mental health difficulties, global subjective distress and impact on health. Random effects linear regression models showed that family connection, self-esteem, problem solving and peer support were negatively associated with all the mental health outcomes.

**Conclusions:**

The findings suggest that the SRS is a valid measure assessing these relevant protective factors, thereby serving as a valuable tool in resilience and mental health research.

**Electronic supplementary material:**

The online version of this article (doi:10.1186/s13034-016-0132-5) contains supplementary material, which is available to authorized users.

## Background

Over the past two decades, there has been a substantial increase in resilience research [1, 2], following dissatisfaction with ‘deficit’ models of illness and psychopathology [3]. Resilience is defined as the maintenance of positive adjustment in the context of exposure to significant adversity [4]. Key protective factors that confer resilience include positive individual characteristics, functional family relationships and a supportive environment outside the family [5, 6]. Individual characteristics such as self-control, empathy, intelligence, self-esteem and problem-solving skills have been identified as beneficial whether someone is facing low or high adversity [e.g. 7]. Similarly, warm relationships within the family and well-structured home environments are important for positive development in all children even in the absence of exposure to stressful life events. However, having a supportive family has been shown to be particularly important for children trying to cope with stressful experiences [e.g. 8]. Lastly, supportive environments outside the family such as availability of social support, school connectedness, having good neighbours and positive role models have been identified as potential protective factors [e.g. 5].

While resilience is conceived of as an end product of buffering processes that do not eliminate risks and stress but allow the individual to deal with them effectively, protective factors have been viewed as moderators of risk and adversity that enhance positive (i.e. developmentally appropriate) outcomes [4, 9]. The measurement of a range of factors that promote positive outcomes is crucial to explore the mechanisms between protective factors and risk factors, and to design intervention and prevention strategies. The student resilience survey [SRS; 10] covers a range of external supports and internal characteristics which can potentially be viewed as protective factors. It was constructed by combining elements from two surveys: the California Healthy Kids Survey [11] and the Perceptions of Peer Support Scale [12], assessing student perceptions of their individual characteristics, protective resources from family, peer, school and community.

The initial SRS development study by Sun and Stewart has supported the validity of the scale [10]. However, there were several limitations to the SRS validation. Firstly, the samples of children were drawn from only 20 primary schools in the state of Queensland, Australia. Sampling from a larger selection of schools, and a wider geographical area, is clearly needed for a more robust assessment of the psychometric properties of this measure. Secondly, although the initial validation study comprised a large sample size (n = 2794), this study will further provide confirmation by including reports from over 7000 children. Thirdly, SRS has only been validated using a scale-level approach. Scale-level analysis does not account for how individuals at different levels of the latent construct perform on the individual items of an instrument [13]. Item-level approaches allow examination of how individual subject responses on items of an instrument relate to an unobservable trait [13]. Differential item functioning [DIF; 14] allows for investigating item response probability based on different groups. DIF is present when individuals from different sociodemographic groupings (such as gender or ethnicity) have a different probability of answering an item [15]. Lastly, the initial validation did not investigate the association between SRS subscales and mental health outcomes. It is expected that most of the SRS subscale scores will be negatively correlated with emotional and behavioural problems [e.g. 16, 17] and attainment of good health [18].

The SRS can be an important tool in assessing the impact of protective factors when investigating the relationship between risk and psychological outcome and development. The purposes of the present study were threefold. First, we aimed to replicate the psychometric characteristics of the SRS found in its initial investigation, this time with an English sample [10]. Second, we aimed to investigate the measurement invariance in regard to several subgroups. Third, we aimed to assess the relationships between the SRS domains and children’s mental health outcomes.

## Methods

### Sample

Data were collected in 2015 from children who were part of a large project that focused on the promotion of resilience and emotional wellbeing (‘HeadStart’, funded by the Big Lottery Fund) in 12 local areas across 90 schools, England. The analyses reported are based on surveys completed by 7663 pupils (42.3% male); 1967 pupils were in primary school (year 6, mean age = 11.38, SD = 0.29) and 5696 pupils were in secondary school (years 7, 8 and 9, mean age = 13.31, SD = 0.86). For the item-level DIF analysis all items needed to be complete, hence only pupils who completed all items were included (sample size ranged from 6047 to 6123).

The sample was not drawn to be representative of all school children in England; it was based on local areas that were part of the HeadStart programme and each of the 12 local areas selected the schools to participate [19]. Overall, 5496 (72.8%) of pupils were White British (compared to the national average of 76.2%), and 6176 (81.6%) pupils’ first language was English (compared to the national average of 82.5%). 1452 (19.1%) were eligible for free school meals (compared to the national average of 16.2%—including nursery schools), 131 (1.7%) had a statement of special educational needs^1^ (compared to the national average of 2.8%) and a further 1159 (15.1%) had any elevated special educational needs, albeit not great enough to meet the threshold for a full statement of SEN.

### Measures

#### Student resilience survey (SRS)

The SRS is a 47-item measure comprising 12 subscales measuring students’ perceptions of their individual characteristics as well as protective factors embedded in the environment. Frequency of each item was rated on a 5-point scale (1 = never to 5 = always).

As this was part of a larger project and it was important not to burden the pupils with a long survey, only 10 of the SRS subscales (out of 12) were selected to be included into the survey with other validated measures. The 10 chosen subscales were: family connection, school connection, community connection, participation in home and school life, participation in community life, peer support, self-esteem, empathy, problem solving, and goals and aspirations. As the main project was interested in identifying protective factors in a child’s life, the pro-social peers (two items: my friends try to do what is right; my friends do well in school) and communication and cooperation (three items: I help other people; I enjoy working with other students; I stand up for myself) subscales were not included. Moreover, the scale was adapted to English school children based on discussions with young advisors from Common Room (a young people’s advocacy and engagement group with a specific focus on disability, health and mental health). Four items were edited so that they were more general and suitable for school-aged children in England (i.e. instead of “are there students at your school who would ask you to play when you are all alone”, it has been changed to “are there students at your school who would ask you to join in when you are all alone”; instead of “are there students at your school who would help you if you hurt yourself in the playground”, it has been changed to “are there students at your school who would help you if you hurt yourself”; instead of “are there students at your school who would invite you to play at their home”, it has been changed to “are there students at your school who would invite you to their home”; instead of “are there students at your school who would share things like stickers, toys & games with you”, it has been changed to are there students at your school who would share things with you”). Lastly, one item from the peer support scale (tell you you’re good at things) was omitted.

*Mental health difficulties* were measured with the me and my feelings questionnaire (formerly known as Me and My School measure, M&MS). It is a 16-item measure comprising a 10-item emotional difficulties scale and a 6-item behavioural difficulties scale [20, 21]. Each item includes a short statement (e.g. I am lonely; I get angry) measured on a 3-point Likert scale (0 = never, 1 = sometimes, and 2 = always) (emotional problems sum score mean = 5.17, SD = 3.87; behavioural problems sum score mean = 3.05, SD = 2.52). Cronbach’s *α*s in the current sample were 0.84 for emotional problems (n = 7187) and 0.80 for behavioural problems (n = 7243).

*Global subjective distress* was measured with child outcome rating scale (CORS). CORS consists of four items: how am I doing; how are things in my family; how am I doing at school; and how is everything going. The rating scale is a 10 cm line with a happy face at one end and a sad face at the other; children are asked to put a mark on the line to indicate the place that best describes how they feel. The score for each item is automatically recorded and the overall score can range from 0 to 40 (sum score mean = 9.59, SD = 7.7); higher scores indicate more global subjective distress [22]. Cronbach’s *α* in the current sample was 0.81 (n = 7448).

*Impact of health on daily life* was measured with the EQ 5D-Y [23]. It has five dimensions: mobility (‘walking about’), self-care (‘looking after myself’), usual activities (‘doing usual activities’), pain and discomfort (‘having pain or discomfort’) and anxiety and depression (‘feeling worried, sad or unhappy’). All items refer to the health state ‘today’. Each item has three levels of problems reported (1 = no problems, 2 = some problems and 3 = a lot of problems) (sum score mean = 6.20, SD = 1.46). Cronbach’s *α* in the current sample was 0.65 (n = 7038).

*Health today* was also measured using the EQ 5D-Y. It included a visual analogue scale where the children rated their overall health status on a scale from 0 to 100 with 0 representing the worst and 100 representing the best health state they can imagine (on that day). In the current study, it was recoded so that higher scores indicated worse health (sum score mean = 20.64, SD = 19.8).

*Special educational needs* (SEN), eligibility for *free school meals* (FSM), and *English as an additional language* (EAL) were derived from the national pupil database (NPD). SEN were based on the school’s assignment of a child to a level of special educational needs. Children with SEN, whether with or without statement, were considered as having special educational needs. FSM is frequently used as an indicator of low family income since only families on income support are entitled to claim free school meals. Lastly, EAL was coded as present if a child’s first language was not English.

### Procedure

Ethical approval was obtained from the University College London Research Ethics Committee. Children completed questionnaires using a secure online system during their usual school day with parent consent. Before pupils responded to the survey, teachers read an information sheet to them which highlighted confidentiality of their answers as well as their right to withdraw from the study. Children provided informed consent prior to completing the survey. The online system was designed to be easy to read and child friendly.

### Analyses

The structure and psychometric properties of the SRS were investigated in several stages. Firstly, confirmatory factor analysis (CFA) was conducted, using Mplus version 7.11 [24], to confirm whether constructs identified as subscales in previous research of this measure are evident in the current sample. This analysis was controlled for intra-class correlation due to clustering by schools [25]. Secondly, differential item functioning (DIF) was investigated across a range of demographic groupings using the Mantel–Haenszel procedure and the Liu–Agresti common log odds ratio as a measure of effect size [26] in DIFAS 5.0 [27]. Thirdly, DTF was conducted to examine the measurement invariance directly at the scale level across different subgroups in DIFAS 5.0. Fourthly, Cronbach’s *α* and McDonald’s *ω* were calculated, using SPSS version 21 and R, to assess the reliability of the subscales. Fifthly, to identify the association between protective factors and mental health outcomes, correlations were run between the SRS subscales and mental health outcomes using SPSS version 21. Lastly, to investigate whether internal or external factors had an impact on mental health outcomes, all subscales of the SRS were entered into regression models at the same time predicting each of the health outcomes. Both unadjusted and adjusted (adjusted for gender, school level—primary/secondary—SEN, EAL and FSM) random effects linear regression analyses (allowing for different school intercepts) were run using STATA version 12; unstandardized Bs, standard error and p-values are reported.

## Results

### Factor structure

Confirmatory factor analysis for ordinal data with weighted least squares with robust standard errors, mean, and variance adjusted (WLSMV) estimator [28] was carried out by testing a model with 10 correlated factors indicated by previous research (Table 1). Given the large sample size, Chi-square was not used to test model fit [29]. Other fit indices (CFI = 0.99; TLI = 0.99; RMSEA = 0.01, SRMR within = 0.03; n = 7663) indicated a good model fit based on widely accepted criteria [30]. The correlation between 10 latent factors ranged between 0.26 and 0.77 (Table 2).

**Table 1:** CFA standardised loadings, measurement errors and intra-class correlations (by school)

Subscales	Items in questionnaire	Factor loading	Measurement error	Intraclass correlation
Family connection	At home, there is an adult who:			
	Is interested in my school work	0.77	0.007	0.040
	Believes that I will be a success	0.85	0.006	0.034
	Wants me to do my best	0.86	0.009	0.057
	Listens to me when I have something to say	0.81	0.005	0.037
School connection	At school, there is an adult who:			
	Really cares about me	0.79	0.024	0.176
	Tells me when I do a good job	0.86	0.011	0.140
	Listens to me when I have something to say	0.84	0.006	0.147
	Believes that I will be a success	0.85	0.010	0.114
Community connection	Away from school, there is an adult who:			
	Really cares about me	0.90	0.003	0.041
	Tells me when I do a good job	0.92	0.002	0.038
	Believes that I will be a success	0.94	0.002	0.040
	I trust	0.85	0.004	0.044
Participation in home and school life	Home and school			
	I do things at home that make a difference (i.e. make things better)	0.77	0.005	0.032
	I help my family make decisions	0.75	0.005	0.014
	At school, I decide things like class activities or rules	0.69	0.007	0.036
	I do things at my school that make a difference (i.e. make things better)	0.81	0.005	0.044
Participation in community life	Away from school			
	I am a member of a club, sports team, church group, or other group	0.80	0.012	0.063
	I take lessons in music, art, sports, or have a hobby	0.88	0.011	0.057
Self-esteem	I can work out my problems	0.77	0.005	0.033
	I can do most things if I try	0.83	0.005	0.061
	There are many things that I do well	0.83	0.005	0.064
Empathy	I feel bad when someone gets their feelings hurt	0.80	0.007	0.043
	I try to understand what other people feel	0.87	0.006	0.036
Problem solving	When I need help, I find someone to talk to	0.83	0.004	0.043
	I know where to go for help when I have a problem	0.84	0.004	0.056
	I try to work out problems by talking about them	0.81	0.004	0.040
Goals and aspirations	I have goals and plans for future	0.75	0.007	0.039
	I think I will be successful when I grow up	0.90	0.006	0.065
Peer support	Are there students at your school who would:			
	Choose you on their team at school	0.72	0.005	0.029
	Explain the rules of a game if you didn’t understand them	0.75	0.005	0.054
	Invite you to their home	0.75	0.004	0.041
	Share things with you	0.83	0.004	0.038
	Help you if you hurt yourself	0.84	0.005	0.050
	Miss you if you weren’t at school	0.79	0.004	0.040
	Make you feel better if something is bothering you	0.86	0.004	0.028
	Pick you for a partner	0.81	0.004	0.034
	Help you if other students are being mean to you	0.85	0.004	0.039
	Tell you you’re their friend	0.87	0.004	0.031
	Ask you to join in when you are all alone	0.86	0.003	0.039
	Tell you secrets	0.73	0.005	0.047

All factor loadings in CFA are significant at p < 0.001; CFI = 0.99; TLI = 0.99; RMSEA = 0.01, SRMR within = 0.03; SRMR between = 0.62; n = 7663

**Table 2:** Factor correlations

	1	2	3	4	5	6	7	8	9	10
1. Family connection	1									
2. School connection	0.55	1								
3. Community connection	0.74	0.56	1							
4. Participation in home and school life	0.60	0.51	0.54	1						
5. Participation in community life	0.30	0.26	0.31	0.42	1					
6. Self-esteem	0.58	0.50	0.54	0.74	0.41	1				
7. Empathy	0.45	0.41	0.42	0.58	0.27	0.53	1			
8. Problem solving	0.56	0.56	0.53	0.70	0.29	0.70	0.60	1		
9. Goals and aspirations	0.55	0.45	0.51	0.62	0.40	0.77	0.46	0.63	1	
10. Peer support	0.53	0.43	0.50	0.55	0.32	0.59	0.52	0.61	0.48	1

All factor correlations are significant at p < 0.0001 level. CFI = 0.99; TLI = 0.99; RMSEA = 0.01, SRMR within = 0.03; SRMR between = 0.62; n = 7663

### Reliability

Since Cronbach’s *α* as a single measure for reliability is no longer regarded as optimal [31], McDonald’s *ω* was also used. Coefficient ω gives a better estimate of reliability than Cronbach’s α if the items of a scale are not tau equivalent [32, 33]. McDonald’s *ωs* were determined using the factor loadings of the multilevel confirmatory factor analysis (within-school factor models; only for the subscales with more than 2 items). The internal consistency for all the subscales was good. Cronbach’s *α* was 0.80 and McDonald’s *ω* was 0.89 (n = 7360) for the family connection subscale; *α* was 0.89 and *ω* was 0.91 (n = 7332) for the school connection subscale; *α* was 0.91 and *ω* was 0.94 (n = 7286) for the community connection subscale; *α* was 0.79 and *ω* was 0.84 (n = 7288) for the participation in home and school life subscale; *α* was 0.74 (n = 7304) for the participation in community life subscale; *α* was 0.80 and *ω* was 0.85 (n = 7358) for the self-esteem subscale; *α* was 0.77 (n = 7391) for the empathy subscale; *α* was 0.83 and *ω* was 0.87 (n = 7314) for the problem-solving subscale; *α* was 0.73 (n = 7324) for the goals and aspirations subscale; lastly *α* was 0.93 and *ω* was 0.96 (n = 7052) for the peer support subscale.

### Differential item functioning (DIF) and differential test functioning (DTF)

In order to examine whether items behaved equivalently across a range of different subgroups of children, DIF analyses were undertaken for all subscales with more than two items. The non-parametric Mantel–Haenszel procedure was chosen to test for DIF since it is not based on the assumptions of a specific item response model [34]. Nevertheless, the subscales were checked to be sufficiently unidimensional based on a single factor multi-level CFA and which was acceptable according to standard criteria for all subscales and only mild violations for ‘participation in home and school life’ were found (see Additional file 1: Table S1 for details). Further, whether the CFA model’s thresholds were ordered along the latent continuum was inspected. Higher item categories corresponded to higher trait levels and only the space on the latent trait corresponding to category 2 was for some items comparatively small [35] (see Additional file 2: Table S2 for item thresholds).

In the DIF analysis, six grouping criteria were examined: gender, primary/secondary school level, whether the child had any elevated special educational need (SEN), whether English was the child’s second language (EAL), and whether the child was eligible for free school meals (FSM). Boys (42.3%, n = 2591), secondary school children (75.0%, n = 4592), children with SEN with or without statement (18.6%, n = 950), non-native English speakers (17.6%, n = 1064), and children receiving FSM (18.4%, n = 1116) were the focus of these investigations (and formed the focal group in DIF analyses). DIF analyses compare the item endorsement rates in the focal group compared to reference group (e.g. children with SEN with or without statement compared to all other children), conditioning on test scores. If children with the same overall score on a subscale (e.g. overall family connection subscale score) have different probabilities of endorsing an individual item then this item is said to show differential item functioning; in other words the item behaves differently across the two groups.

The Liu-Agresti common logs ratio [L-A-LOR; 26] was used to assess the size of potential DIF effects and to gauge their potential relevance. Positive L-A-LOR values indicate the item is more difficult to endorse for the focal group, while negative L-A-LOR values indicate that the item is more difficult to endorse for the reference group, given the same level of underlying trait. The magnitude of DIF was interpreted using a widely accepted classifying system [36]: the magnitude was negligible if L-A-LOR was less than 0.43, moderate if L-A-LOR was between 0.43 and 0.64, and large if L-A-LOR was greater than 0.64.

According to the DIF analysis (Table 3), boys were more likely to agree that students at school were more likely to pick them for a partner and girls were more likely to agree that students told them their secrets and missed them if they weren’t at school. Primary school children were more likely to agree that they do things at school which make a difference; that their peers explain to them the rules if they don’t understand, and that their peers help them if they hurt themselves. Secondary school children were more likely than primary school children to endorse that there are students at their school who would tell them their secrets. Children with SEN were more likely to agree that their family listens to them when they have something to say. Lastly, children without EAL were more likely to agree that peers invite them to their home. Analyses did not indicate any DIF based FSM eligibility.

**Table 3:** Differential item functioning and differential test functioning for the resilience scale

Subscales	Items in questionnaire	Gender (focal = boys) n = 6123		School level (focal = secondary) n = 6123		SEN (focal = yes) n = 6071		EAL (focal = yes) n = 6047		FSM (focal = yes) n = 6071	
		DIF	DTF	DIF	DTF	DIF	DTF	DIF	DTF	DIF	DTF
		L-A LOR (SE)	v2 (SE)	L-A LOR (SE)	v2 (SE)	L-A LOR (SE)	v2 (SE)	L-A LOR (SE)	v2 (SE)	L-A LOR (SE)	v2 (SE)
Family connection	At home, there is an adult who…										
	Is interested in my school work	0.24 (0.07)	0.03 (0.03)	−0.15 (0.07)	0.03 (0.03)	0.15 (0.09)	0.09 (0.07)	−0.04 (0.09)	0.02 (0.02)	0.29 (0.08)	0.03 (0.03)
	Believes that I will be a success	−0.10 (0.07)		0.15 (0.08)		0.27 (0.10)		0.25 (0.09)		-0.14 (0.10)	
	Wants me to do my best	0.17 (0.11)		0.34 (0.13)		0.25 (0.14)		−0.19 (0.14)		0.14 (0.13)	
	Listens to me when I have something to say	−0.22 (0.07)		−0.08 (0.07)		−*0.48 (0.09)*		−0.08 (0.09)		−0.24 (0.08)	
School connection	At school, there is an adult who…										
	Really cares about me	0.04 (0.06)	0.000 (0.003)	0.37 (0.07)	0.05 (0.04)	−0.38 (0.09)	0.05 (0.04)	0.08 (0.08)	0.01 (0.01)	−0.16 (0.08)	0.01 (0.01)
	Tells me when I do a good job	0.03 (0.06)		−0.02 (0.07)		0.28 (0.09)		−0.21 (0.08)		0.09 (0.08)	
	Listens to me when I have something to say	−0.11 (0.06)		−0.18 (0.08)		0.03 (0.09)		0.04 (0.08)		0.18 (0.08)	
	Believes that I will be a success	0.04 (0.06)		−0.23 (0.08)		0.12 (0.09)		0.08 (0.08)		−0.09 (0.08)	
Community connection	Away from school, there is an adult who…										
	Really cares about me	0.39 (0.08)	0.05 (0.04)	−0.15 (0.09)	−0.001 (0.01)	−0.05 (0.11)	−0.01 (0.004)	0.19 (0.11)	0.01 (0.02)	−0.05 (0.10)	−0.01 (0.003)
	Tells me when I do a good job	−0.10 (0.08)		0.00 (0.09)		−0.08 (0.10)		−0.21 (0.10)		−0.01 (0.10)	
	Believes that I will be a success	0.04 (0.08)		0.05 (0.09)		0.11 (0.11)		0.11 (0.10)		−0.07 (0.10)	
	I trust	−0.23 (0.08)		0.07 (0.09)		0.02 (0.10)		−0.05 (0.10)		0.10 (0.10)	
Participation in home and school life	I do things at home that make a difference (i.e. make things better)	−0.07 (0.06)	0.001 (0.003)	−0.05 (0.06)	0.10 (0.07)	0.13 (0.08)	0.001 (0.01)	−0.11 (0.07)	0.02 (0.02)	−0.01 (0.07)	0.01 (0.01)
	I help my family make decisions	−0.03 (0.06)		−0.22 (0.06)		−0.10 (0.08)		0.27 (0.07)		−0.14 (0.07)	
	At school, I decide things like class activities or rules	−0.01 (0.05)		−0.22 (0.06)		0.01 (0.07)		−0.06 (0.07)		−0.02 (0.07)	
	I do things at my school that make a difference (i.e. make things better)	0.10 (0.06)		*0.56 (0.06)*		−0.04 (0.08)		−0.12 (0.07)		0.19 (0.07)	
Self-esteem	I can work out my problems	−0.10 (0.06)	0.03 (0.02)	−0.35 (0.07)	0.07 (0.06)	0.17 (0.08)	0.01 (0.02)	0.21 (0.08)	0.02 (0.02)	0.18 (0.08)	0.01 (0.02)
	I can do most things if I try	0.26 (0.07)		0.23 (0.07)		−0.15 (0.09)		−0.11 (0.09)		−0.09 (0.08)	
	There are many things that I do well	−0.11 (0.06)		0.21 (0.07)		−0.07 (0.08)		−0.16 (0.08)		−0.13 (0.08)	
Problem solving	When I need help, I find someone to talk to	0.36 (0.06)	0.06 (0.05)	0.08 (0.07)	0.004 (0.01)	0.04 (0.08)	−0.01 (0.001)	−0.21 (0.08)	0.02 (0.02)	0.01 (0.08)	0.003 (0.01)
	I know where to go for help when I have a problem	−0.20 (0.06)		0.06 (0.07)		−0.01 (0.09)		0.11 (0.09)		−0.13 (0.08)	
	I try to work out problems by talking about them	−0.15 (0.06)		−0.13 (0.07)		−0.03 (0.09)		0.10 (0.08)		0.11 (0.08)	
Peer support	Are there students at your school who would…										
	Choose you on their team at school	−0.40 (0.06)	0.12 (0.05)	0.19 (0.06)	0.13 (0.06)	0.26 (0.08)	0.04 (0.02)	−0.10 (0.07)	0.07 (0.03)	0.10 (0.07)	0.004 (0.004)
	Explain the rules of a game if you didn’t understand them	−0.26 (0.06)		*0.53 (0.07)*		−0.10 (0.08)		−0.40 (0.08)		−0.10 (0.08)	
	Invite you to their home	−0.17 (0.06)		−0.32 (0.06)		0.25 (0.08)		*0.75 (0.08)*		0.20 (0.07)	
	Share things with you	0.01 (0.06)		−0.28 (0.07)		0.05 (0.08)		−0.13 (0.08)		0.07 (0.08)	
	Help you if you hurt yourself	−0.03 (0.07)		*0.76 (0.08)*		−0.27 (0.09)		−0.27 (0.09)		0.01 (0.08)	
	Miss you if you weren’t at school	*0.76 (0.06)*		−0.23 (0.06)		−0.16 (0.08)		−0.01 (0.08)		0.03 (0.07)	
	Make you feel better if something is bothering you	0.28 (0.06)		0.24 (0.07)		−0.29 (0.08)		0.08 (0.08)		-0.13 (0.08)	
	Pick you for a partner	−*0.45 (0.07)*		−0.14 (0.07)		0.33 (0.08)		0.04 (0.09)		0.02 (0.08)	
	Help you if other students are being mean to you	−0.03 (0.06)		0.16 (0.07)		−0.17 (0.08)		−0.12 (0.08)		−0.14 (0.08)	
	Tell you you’re their friend	−0.07 (0.07)		0.16 (0.08)		−0.07 (0.09)		0.03 (0.09)		−0.08 (0.09)	
	Ask you to join in when you are all alone	−0.34 (0.07)		−0.02 (0.07)		−0.28 (0.09)		−0.29 (0.09)		−0.08 (0.08)	
	Tell you secrets	*0.45 (0.06)*		−*0.62 (0.06)*		0.20 (0.08)		0.08 (0.08)		−0.02 (0.07)	

*EAL* English as an additional language, *FSM* free school meals, *SEN* special educational needs. DIF and DTF analyses for subscales with less than 3 items (participation in community life, empathy, and goals and aspirations) are not conducted. The size of the DIF is considered negligible if L-A-LOR < 0.43, moderate if between 0.43 and 0.64, and large if >0.64. The size of the DTF ν^2^ < 0.07 indicates small DTF, 0.07 ≤ ν^2^ ≤ 0.14 indicates medium DTF, and ν^2^ > 0.14 indicates large DTF

Differential test functioning (DTF) assesses the aggregate effect of DIF across all the items in a scale [37]. A scale with a DIF effect variance of ν^2^ below 0.07 can be classified as having small DTF, whereas DTF would be considered medium for 0.07 ≤ ν^2^ ≤ 0.14 and large for ν^2^ > 0.14 [37]. All subscales, except the peer support subscale, showed small DTF whereas the peer support subscale showed moderate DTF (Table 3).

### The student resilience survey and mental health problems

A pattern was identified whereby the subscales of the SRS are negatively associated with mental health difficulties, global subjective distress and impact on health (Table 4). In terms of negligible associations, empathy demonstrated very low correlations with both emotional problems and health. On the other hand, both self-esteem and problem solving had moderate correlations with emotional problems and global subjective distress.

**Table 4:** Correlations between student resilience subscales and other scales

	Emotional problems	Behavioural problems	Global subjective distress	Impact of health on daily life	Health today (0–100)
Family connection	−.29* (n = 6944)	−.34* (n = 6995)	−.41* (n = 7160)	−.31* (n = 6775)	−.30* (n = 7033)
School connection	−.22* (n = 6911)	−.26* (n = 6963)	−.39* (n = 7134)	−.21* (n = 6752)	−.25* (n = 7013)
Community connection	−.29* (n = 6876)	−.25* (n = 6926)	−.36* (n = 7082)	−.27* (n = 6717)	−.28* (n = 6967)
Participation in home and school life	−.32* (n = 6903)	−.29* (n = 6958)	−.40* (n = 7089)	−.26* (n = 6753)	−.27* (n = 7002)
Participation in community life	−.15* (n = 6882)	−.10* (n = 6932)	−.16* (n = 7101)	−.11* (n = 6736)	−.19* (n = 6987)
Self-esteem	−.44* (n = 6969)	−.32* (n = 7018)	−.49* (n = 7157)	−.35* (n = 6816)	−.36* (n = 7072)
Empathy	−.07* (n = 7004)	−.27* (n = 7045)	−.21* (n = 7189)	−.10* (n = 6847)	−.17* (n = 7105)
Problem solving	−.37* (n = 6933)	−.33* (n = 6980)	−.44* (n = 7112)	−.30* (n = 6778)	−.29* (n = 7038)
Goals and aspirations	−.32* (n = 6939)	−.23* (n = 6980)	−.37* (n = 7122)	−.25* (n = 6787)	−.28* (n = 7042)
Peer support	−.36* (n = 6681)	−.24* (n = 6725)	−.35* (n = 6855)	−.31* (n = 6533)	−.27* (n = 6772)

* p < 0.0001

Furthermore, in order to investigate whether internal or external factors had an impact on mental health outcomes, all subscales of the SRS were entered into linear regression models (with school as a random effects term) at the same time predicting each of the health outcomes (Table 5). The adjusted regression results showed that family connection, self-esteem, problem solving and peer support were negatively associated with all the mental health outcomes. On the other hand, those who had high empathy were more likely to display mental health difficulties, global subjective distress and impact on health.

**Table 5:** Unadjusted and adjusted associations between student resilience survey subscales and other scales

Unadjusted analysis	Emotional problems (n = 5561)		Behavioural problems (n = 5595)		Global subjective distress (n = 5655)		Impact of health on daily life (n = 5439)		Health today (0–100) (n = 5621)	
Student resilience survey subscales	Estimate (SE)	P	Estimate (SE)	P	Estimate (SE)	P	Estimate (SE)	P	Estimate (SE)	P
Fixed effects										
Intercept	*15.06 (0.34)*	*<.0001*	9.72 (0.23)	*<.0001*	*36.07 (0.65)*	*<.0001*	*9.84 (0.14)*	*<.0001*	*71.78 (1.81)*	*<.0001*
Family connection	*−0.08 (0.02)*	*<.0001*	*−0.16 (0.01)*	*<.0001*	*−0.46 (0.04)*	*<.0001*	*−0.07 (0.01)*	*<.0001*	*−0.83 (0.11)*	*<.0001*
School connection	0.02 (0.01)	0.216	*−0.03 (0.01)*	*<.0001*	*−0.20 (0.03)*	*<.0001*	−0.00 (0.01)	0.773	**−** *0.22 (0.07)*	*0.003*
Community connection	*−0.06 (0.02)*	*0.001*	−0.01 (0.01)	*0.563*	*−0.12 (0.03)*	*<.0001*	*−0.02 (0.01)*	*0.009*	*−0.20 (0.09)*	*0.026*
Participation in home and school life	−0.03 (0.02)	0.095	*−0.02 (0.01)*	*0.040*	*−0.08 (0.03)*	*0.012*	0.01 (0.01)	0.217	0.00 (0.09)	0.992
Participation in community life	−0.01 (0.02)	0.516	*0.03 (0.01)*	*0.024*	0.05 (0.03)	0.112	0.00 (0.01)	0.493	*−0.39 (0.09)*	*<.0001*
Self-esteem	*−0.42 (0.03)*	*<.0001*	*−0.14 (0.02)*	*<.0001*	*−0.80 (0.05)*	*<.0001*	*−0.12 (0.01)*	*<.0001*	*−1.46 (0.14)*	*<.0001*
Empathy	*0.52 (0.03)*	*<.0001*	*−0.12 (0.02)*	*<.0001*	*0.48 (0.05)*	*<.0001*	*0.12 (0.01)*	*<.0001*	*0.38 (0.15)*	*0.012*
Problem solving	*−0.22 (0.02)*	*<.0001*	*−0.10 (0.01)*	*<.0001*	*−0.35 (0.04)*	*<.0001*	*−0.05 (0.01)*	*<.0001*	*−0.36 (0.10)*	*<.0001*
Goals and aspirations	*−0.10 (0.03)*	*0.001*	*0.04 (0.02)*	*0.050*	*−0.13 (0.06)*	*0.029*	−0.01 (0.01)	*0.460*	*−0.37 (0.16)*	*0.024*
Peer support	*−0.06 (0.01)*	*<.0001*	*0.01 (0.00)*	*0.013*	*−0.04 (0.01)*	*0.001*	**−** *0.02 (0.00)*	*<.0001*	*0.12 (0.03)*	*<.0001*
Variance components										
Residual variance	3.21 (0.03)		2.24 (0.02)		6.11 (0.06)		1.30 (0.01)		17.49 (0.17)	
School-level	0.72 (0.09)		0.38 (0.06)		0.92 (0.14)		0.23 (0.04)		2.22 (0.43)	

Adjusted analysis^a^	Emotional problems (n = 5488)		Behavioural problems (n = 5522)		Global subjective distress (n = 5584)		Impact of health on daily life (n = 5371)		Health today (0–100) (n = 5548)	
Student resilience survey subscales	Estimate (SE)	P	Estimate (SE)	P	Estimate (SE)	P	Estimate (SE)	P	Estimate (SE)	P
Fixed effects										
Intercept	*18.87 (0.46)*	*<.0001*	*8.87 (0.31)*	*<.0001*	*36.29 (0.87)*	*<.0001*	*10.55 (0.19)*	*<.0001*	*70.66 (2.46)*	*<.0001*
Family connection	*−0.08 (0.02)*	*<.0001*	*−0.16 (0.01)*	*<.0001*	*−0.47 (0.04)*	*<.0001*	*−0.07 (0.01)*	*<.0001*	*−0.83 (0.11)*	*<.0001*
School connection	0.01 (0.01)	0.476	*−0.04 (0.01)*	*<.0001*	*−0.20 (0.03)*	*<.0001*	−0.01 (0.01)	*0.245*	*−0.23 (0.08)*	*0.002*
Community connection	*−0.06 (0.02)*	*<.0001*	−0.01 (0.01)	0.625	*−0.13 (0.03)*	*<.0001*	*−0.02 (0.01)*	*0.008*	−0.17 (0.09)	0.052
Participation in home and school life	−0.03 (0.02)	0.055	*−0.03 (0.01)*	*0.014*	*−0.09 (0.03)*	*0.005*	0.01 (0.01)	0.420	−0.04 (0.09)	0.630
Participation in community life	0.01 (0.02)	0.640	*0.03 (0.01)*	*0.011*	*0.07 (0.03)*	*0.036*	0.01 (0.01)	0.183	*−0.31 (0.09)*	*0.001*
Self-esteem	*−0.36 (0.03)*	*<.0001*	*−0.14 (0.02)*	*<.0001*	*−0.76 (0.05)*	*<.0001*	*−0.11 (0.01)*	*<.0001*	*−1.37 (0.14)*	*<.0001*
Empathy	*0.45 (0.03)*	*<.0001*	*−0.09 (0.02)*	*<.0001*	*0.44 (0.05)*	*<.0001*	*0.11 (0.01)*	*<.0001*	*0.31 (0.15)*	*0.047*
Problem solving	*−0.20 (0.02)*	*<.0001*	*−0.10 (0.01)*	*<.0001*	*−0.34 (0.04)*	*<.0001*	*−0.05 (0.01)*	*<.0001*	*−0.32 (0.10)*	*0.001*
Goals and aspirations	*−0.07 (0.03)*	*0.024*	0.03 (0.02)	0.114	−0.09 (0.06)	0.109	−0.01 (0.01)	0.594	*−0.37 (0.16)*	*0.024*
Peer support	*−0.08 (0.01)*	*<.0001*	*0.02 (0.00)*	*<.0001*	**−** *0.05 (0.01)*	*<.0001*	*−0.02 (0.00)*	*<.0001*	*−0.13 (0.03)*	*<.0001*
Variance components										
Residual variance	3.15 (0.03)		2.22 (0.21)		6.07 (0.06)		1.29 (0.01)		17.42 (0.17)	
School-level	0.54 (0.08)		0.27 (0.06)		0.79 (0.15)		0.18 (0.04)		2.03 (0.44)	

Each column represents a new regression model. All models were tested using multilevel linear regression

^a^Adjusted analysis are controlling for gender, school level (primary/secondary), special education needs, English as an additional language and free school meals

## Discussion

The aim of this study was to replicate the psychometric properties of the student resilience survey (SRS) within an English sample, to investigate the measurement invariance in subgroups, and to investigate the relationship between SRS subscales and mental health.

On the basis of the confirmatory factor analysis, the factor structure of the measure was similar to the original validation study showing that 41 items loaded uniquely onto their respective 10 subscales. Moreover, similar to the validation study, the analyses provided evidence for acceptable reliability of all the subscales for this sample, especially considering that some of them are very short//have very few items//something like that [10]. Nevertheless, the ‘participation in home and school life’ showed in individual analyses that it is not unidimensional and will need further research investigating its structure.

This study extended previous research by generating evidence of the SRS’s validity via analysis of DIF and DTF. Our DIF analysis indicated differences between boys and girls in terms of how they answered the peer support subscale of the SRS. This may be due to the differences between boys’ and girls’ expectations from friends. One study has shown that boys describe friends as “people you play with” whereas girls describe them as “people you can trust” [38]. Moreover, DIF analyses suggested that children in secondary school were more likely to agree that their peers shared secrets with them. This is in line with literature suggesting that during middle childhood, the quality of friendship changes from relationships characterised by the temporary sharing of activities and leisure time to friendships enhanced by intimacy, help, certainty, loyalty and confidence [39]. Primary school children were also more likely to agree that their peers explained the rules of play to them and helped if they hurt themselves. This may be due to children in secondary school becoming more independent and working through their problems more easily than younger children [40]. Children with special educational needs were more likely to agree that their families listened to them when they have something to say. The quality of caregiving by parents is crucial for children with special educational needs [41], hence parents of children with special educational needs might be making an extra effort to engage with their children. Children with English as a first language were more likely to agree that peers invited them to their home. Literature suggests that children prefer same-ethnic friends, even when the opportunity to meet out-group friends has been taken into account [42]. Hence, children with English as a first language might get more invites to friends’ homes. All subscales, except the peer support subscale, showed small DTF whereas the peer support subscale showed moderate DTF. Future research should explore in more detail whether the DIF found is due to qualitative differences and constitutes relevant item and scale bias that needs to be addressed by changing items or separate norming tables (especially for school and gender [43]).

As expected on the basis of related evidence, our results showed negative correlations between all the SRS subscales and emotional, behavioural and health problems. Adjusted random effects linear regression models showed that family connection, self-esteem, problem solving and peer support were negatively associated with emotional, behavioural and health problems. The capacity of supportive families to buffer children from the impact of stressful life events is well documented [44]. For example, family-related factors such as parenting style and parent–child relationship quality have been associated with improved psychosocial adjustment [e.g. 45, 46] and attainment of good health [18]. Having a positive parent–child relationship may translate into an opportunity for parents to guide their children in understanding emotions and dealing with stress. Additionally, it has been shown that healthy peer relationships and healthy school environment can protect youth from maladjustment (i.e. emotional and behavioural problems) [47] and health problems [18]. Similar to previous literature, self-esteem and problem-solving skills have been negatively associated with emotional problems [48, 49]. Problem-solving skills and self-esteem may enable children to alter the source of stress which in return may have a positive impact on mental and physical health problems [50]. On the other hand our results showed that the correlation between empathy and emotional problems was very low. Moreover, when all the subscales were loaded into the random effects regression models predicting mental health outcomes, empathy was positively associated with emotional, behavioural and health problems. The literature suggests that extreme empathy may increase vulnerability to mental health difficulties [51, 52].

In general, protective factors alter responses to adverse events so that potential negative outcomes can be avoided [9]. According to Benzies and Mychasiuk [53], resilience is optimised when protective factors are strengthened at all interactive levels of the socio-ecological model (i.e. individual, family and community). Identifying protective factors that promote resilience to mental health problems could lead to improved intervention strategies [1].

It is important to note the methodological limitations of the study. Firstly, the population of the study was not drawn to be representative of all school children in England; however, the participants were from 12 local areas of England which increased the generalisability of the results. Secondly, due to the overlap between the items of the SRS and the mental health difficulties questionnaires and the cross-sectional nature of this study, we cannot disentangle temporal precedence (what comes first) and causal mechanisms between the resilience factors and mental health outcomes. However, future studies should apply longitudinal methods to elucidate the relationship between the SRS protective factors and maladjustment. Thirdly, this study did not investigate the stability of scores; hence future studies should examine the test–retest reliability of the SRS. Lastly, our approach addressed the clustering due to schools in our sample with an appropriate multilevel approach to identify between-student variation [25]; but we did not model the differences between schools. The corresponding SRMR values for the between-school variance–covariance matrices were consequently bad (SRMR = 0.61 for full model; see also Additional file 1: Table S1). The intra-class correlations presented in Table 1 also show that relevant amounts of variance in item responses were due to differences between schools. Future research should explore school-characteristics to further explore these differences. Additionally, future research may investigate whether the SRS could be effectively or appropriately utilised with children who have clinical levels of mental health problems.

## Conclusions

To the best of our knowledge, this is the first study to investigate the validity of the student resilience survey in the UK and to explore its relationship with mental health and wellbeing. Notwithstanding the limitations, our results showed that SRS subscales have loaded uniquely onto their respective 10-factor structure. All the SRS subscales had high reliability and the subscales (except for empathy) were negatively associated with mental health problems, global subjective distress and impact on health. Given the interest of investigating the relationship between risk, psychological outcomes and development, the SRS provides an exciting possibility to assess several different protective factors, and can thus be used as a valuable measurement tool in resilience and risk factor research.

## Additional files

**Additional file 1: Table S1.** Fit Indices for Univariate Student Resilience Subscales.

**Additional file 2: Table S2.** Unstandardised Factor Loadings (λ) and Thresholds (τ) for the Items of Student Resilience Survey from Unidimensional CFA Models for the Individual Subscales.

## Abbreviations

CFA: confirmatory factor analysis
CORS: child outcome rating scale
DIF: differential item functioning
DTF: differential test functioning
EAL: English as an additional language
FSM: free school meals
M&MS: me and my feelings questionnaire
NPD: national pupil database
SEN: special educational needs
SRS: student resilience survey

## Footnotes

1 A statement of special educational needs is a formal document outlining the nature of a given child’s needs that is produced following a process of statutory assessment (by, for example, an educational psychologist).
