# Supplementary material for: The student resilience survey: psychometric validation and associations with mental health
Source: Child Adolesc Psychiatry Ment Health. 2016 Nov 3;10:44. doi: 10.1186/s13034-016-0132-5 (PMC5093941; doi:10.1186/s13034-016-0132-5)
Supplement: Supplementary file 1 — Additional file 1: Table S1. Fit Indices for Univariate Student Resilience Subscales. [file 13034_2016_132_MOESM1_ESM.docx]

| **Supplementary Table 1: Fit Indices for Univariate Student Resilience Subscales** | | | | | | | |
| --- | --- | --- | --- | --- | --- | --- | --- |
|  | **N** | **RMSEA** | **CFI** | **TLI** | **SRMR** | | **Chi2 (df), p** |
|  |  |  |  |  | **Within** | **Between** |  |
| Family connection | 7656 | 0.024 | 0.999 | 0.998 | 0.009 | 0.626 | 44.617 (8), < 0.001 |
| School connection | 7595 | 0.038 | 0.998 | 0.997 | 0.003 | 0.751 | 95.780 (8), < 0.001 |
| Community connection | 7571 | 0.036 | 1.000 | 0.999 | 0.011 | 0.742 | 85.409 (8), < .0001 |
| Participation in home and school life | 7535 | 0.085 | 0.987 | 0.980 | 0.051 | 0.597 | 446.749 (8), < .0001 |
| Self-esteem | 7517 | 0.034 | 0.999 | 0.998 | 0.000 | 0.642 | 29.627 (3), <.0001 |
| Problem solving | 7486 | 0.039 | 0.999 | 0.998 | 0.000 | 0.667 | 37.647 (3), <.0001 |
| Peer support | 7611 | 0.035 | 0.994 | 0.993 | 0.036 | 0.694 | 1252.097 (120), < .0001 |
| Note: CFI = comparative fit index; RMSEA = root mean square error of approximation; SRMR = standardized root mean square residual; Fit indices for subscales with less than 3 items (participation in community life, empathy, and goals and aspirations) are not reported | | | | | | | |
