# Supplementary material for: The student resilience survey: psychometric validation and associations with mental health
Source: Child Adolesc Psychiatry Ment Health. 2016 Nov 3;10:44. doi: 10.1186/s13034-016-0132-5 (PMC5093941; doi:10.1186/s13034-016-0132-5)
Supplement: Supplementary file 2 — Additional file 2: Table S2. Unstandardised Factor Loadings (λ) and Thresholds (τ) for the Items of Student Resilience Survey from Unidimensional CFA Models for the Individual Subscales. [file 13034_2016_132_MOESM2_ESM.docx]

| Supplementary Table 2: Unstandardised Factor Loadings (λ) and Thresholds (τ) for the Items of Student Resilience Survey from Unidimensional CFA Models for the Individual Subscales | | | | | |
| --- | --- | --- | --- | --- | --- |
| **Items in questionnaire** | **Unstandardized λ** | **τ1** | **τ2** | **τ3** | **τ4** |
| **Family Connection** |  |  |  |  |  |
| Is interested in my school work | 1.00 | -3.12 | -2.34 | -1.18 | -0.15 |
| Believes that I will be a success | 1.22 | -4.01 | -3.32 | -2.16 | -1.05 |
| Wants me to do my best | 1.37 | -5.15 | -4.58 | -3.56 | -2.54 |
| Listens to me when I have something to say | 1.00 | -3.19 | -2.37 | -1.34 | -0.28 |
|  | | | | | |
| **School Connection** |  |  |  |  |  |
| Really cares about me | 1.00 | -3.19 | -2.21 | -0.85 | 0.19 |
| Tells me when I do a good job | 1.19 | -4.14 | -3.09 | -1.70 | -0.18 |
| 7. Listens to me when I have something to say | 1.13 | -3.76 | -2.69 | -1.30 | 0.01 |
| Believes that I will be a success | 1.11 | -3.70 | -2.72 | -1.43 | -0.14 |
|  |  |  |  |  |  |
| **Community connection** |  |  |  |  |  |
| Really cares about me | 1.00 | -4.92 | -4.11 | -2.77 | -1.49 |
| Tells me when I do a good job | 1.01 | -4.88 | -3.86 | -2.37 | -0.75 |
| Believes that I will be a success | 1.21 | -5.88 | -4.76 | -3.09 | -1.36 |
| I trust | 0.70 | -3.45 | -2.82 | -1.98 | -1.10 |
|  |  |  |  |  |  |
| **Participation in home and school life** |  |  |  |  |  |
| I do things at home that make a difference (i.e., make things better) | 1.00 | -2.76 | -2.05 | -0.64 | 0.73 |
| I help my family make decisions | 0.91 | -2.34 | -1.55 | -0.51 | 0.67 |
| At school, I decide things like class activities or rules | 0.94 | -1.33 | -0.47 | 0.63 | 1.58 |
| I do things at my school that make a difference (i.e. make things better) | 1.26 | -2.54 | -1.43 | -0.05 | 1.32 |
|  |  |  |  |  |  |
| **Self-esteem** |  |  |  |  |  |
| I can work out my problems | 1.00 | -2.66 | -1.81 | -0.68 | 0.49 |
| I can do most things if I try | 1.79 | -4.76 | -3.56 | -1.88 | 0.09 |
| There are many things that I do well | 1.35 | -3.69 | -2.62 | -1.19 | 0.38 |
|  |  |  |  |  |  |
| **Problem solving** |  |  |  |  |  |
| When I need help, I find someone to talk to | 1.00 | -2.90 | -1.80 | -0.61 | 0.59 |
| I know where to go for help when I have a problem | 0.81 | -2.93 | -2.07 | -1.03 | -0.11 |
| I try to work out problems by talking about them | 0.89 | -2.23 | -1.37 | -0.27 | 0.83 |
|  |  |  |  |  |  |
| **Peer support** |  |  |  |  |  |
| Choose you on their team at school | 1.00 | -2.38 | -1.74 | -0.74 | 0.26 |
| Explain the rules of a game if you didn’t understand them | 1.02 | -2.85 | -2.18 | -1.18 | -0.16 |
| Invite you to their home | 1.13 | -2.16 | -1.54 | -0.60 | 0.45 |
| Share things with you | 1.44 | -3.38 | -2.51 | -1.30 | 0.12 |
| Help you if you hurt yourself | 1.41 | -3.45 | -2.69 | -1.65 | -0.51 |
| Miss you if you weren’t at school | 1.27 | -2.29 | -1.57 | -0.57 | 0.44 |
| Make you feel better if something is bothering you | 1.51 | -3.41 | -2.47 | -1.37 | -0.17 |
| Pick you for a partner | 1.35 | -3.18 | -2.43 | -1.44 | -0.29 |
| Help you if other students are being mean to you | 1.48 | -3.16 | -2.38 | -1.39 | -0.22 |
| Tell you you’re their friend | 1.65 | -3.85 | -3.07 | -1.95 | -0.70 |
| Ask you to join in when you are all alone | 1.61 | -3.23 | -2.44 | -1.39 | -0.20 |
| Tell you secrets | 1.05 | -1.97 | -1.44 | -0.64 | 0.24 |
| Note: Subscales with less than 3 items (participation in community life, empathy, and goals and aspirations) are not reported | | | | | |
